# Supplementary material for: Telemedicine and its acceptance by patients with type 2 diabetes mellitus at a single care center during the COVID-19 emergency: A cross-sectional observational study
Source: PLoS One. 2023 Feb 15;18(2):e0269350. doi: 10.1371/journal.pone.0269350 (PMC9931089; doi:10.1371/journal.pone.0269350)
Supplement: Attachment — Submitted filename: Response to Reviewers.docx [file pone.0269350.s001.docx]

Response to Editor and Reviewers

We thank the Editor and the Reviewers for careful evaluation of our study and for all their valuable suggestions in order to improve the scientific merit of the manuscript. Detailed answers to all the criticisms are reported here below in red color.

Additional Editor Comments: STROBE diagram needs to be added. There are many grammatical errors that need correction. The discussion needs improvement.

We thank the Editor for suggestion. A STROBE diagram has been included in the revised version of the manuscript. The text was carefully checked. Additional discussion has been provided.

Reviewer #1:

1. sample size could have been increased for better inference.

The issue of sample size was raised also by Reviewer no. 3. Indeed, we conducted a sample size estimation, based on previously published cross-sectional studies on telemedicine, reporting a high degree of satisfaction (Palandri et al., 2020; Riley et al., 2021). With a provisional estimate of 80% of satisfied/very satisfied patients, a precision value of 0.05 and a *Z*-value of 1.96 (at 5% type I error), the formula (Wang & Ji, *Chest,* 158(1), pp.S12-S20, 2020) yielded a sample size of 246 subjects, that was rounded to 250. Therefore, according to the calculations, the number of subjects is within an adequate limit.

This information was included in the manuscript.

2. few grammatical mistakes are there , please correct it.

The text was carefully checked.

3. please clarify , wether during telemedicine patient can see (Video interaction) their physician or not.

Telemedicine consultation consisted of a call that replaced the check-up visit, where the reports sent by e-mail were analyzed and a therapy confirmed or set. This information has been added to the manuscript.

Reviewer #2:

1. This is a single cohort study without a control group during the Covid-19 pandemic when in person services were interrupted due to restrictions.

According to the suggestion of another Reviewer, we corrected the classification of the present study as cross-sectional survey.

2. While methodology is sound the paper needs to be viewed in perspective.

Additional discussion has been provided.

3. This being a simple observational study it is not understood in what way “Standards of Good Clinical Practice and ICH Harmonized Tripartite Guidelines” were applicable in this study. These guidelines are for clinical trials.

We thank the Reviewer for remark. Correction was applied to the description of the methodology.

4. Some care is better than no care. People around the world were numbed during the unprecedented severe restrictions in this period.

A comment referring to this issue was added in the text.

5. In such an environment even tele-consultation would have been welcome. The study findings need to be interpreted against this background.

Additional comment was added in the text.

6. Perhaps another study using same methods but in a post-pandemic setting would complement this study. Can be a recommendation in this paper.

Additional comment was added in the text.

7. Post pandemic tele-consultation lessons learned could be useful for delivering medical care in underserved and remote areas. However, the temptation to overuse it in normal settings should be checked.

Additional comment was added in the text.

8. May consider discussing some of these issues in the paper.

We thank the Reviewer for all these suggestions, that have been acknowledged.

Reviewer #3:

Dear author,

Thank you for undertaking this research to analyse the perceptions of patients on telehealth.

Overall the article is reasonably well presented. However I suggest that the following issues be addressed.

1. The setting needs to be well explained. The nature of the clinical practice (teaching or non teaching clinic), the characteristics of patients that are captured in this clinical practice whether it is a public or a private practice and the population that the clinic covers.

Additional information has been provided in the Methods section.

2. A STROBE diagram seems essential. Ideally it could start from the number of patients that attended the clinic during the study period number of type 2 diabetics, number who underwent tele health consultations and the number of patients who took part in the study. It is interesting that all 250 consecutive participants consented for the study and participated without any issues.

We thank the Reviewer for suggestion. A STROBE diagram has been included in the revised version of the manuscript.

3. Sample size calculation has not been addressed. Starting from the hypothesis of this study, the reason as to how the number 250 was arrived at needs to be described clearly.

Sample size was determined based on previous cross-sectional studies on telemedicine, reporting a high degree of satisfaction (Palandri et al., 2020; Riley et al., 2021). With a provisional estimate of 80% of satisfied/very satisfied patients, a precision value of 0.05 and a *Z*-value of 1.96 (at 5% type I error), the formula (Wang & Ji, *Chest,* 158(1), pp. S12-S20, 2020) yielded a sample size of 246 subjects, that was rounded to 250.

This information was included in the manuscript.

4. Regarding the study design, I disagree if this is a cohort study. There is no follow-up of patients. It seems to be a cross sectional study looking at the perspectives of type 2 diabetics who have attended tele-health consultation

We thank the Reviewer for this suggestion; accordingly, the study design type was corrected in the manuscript.

5. While collecting baseline characteristics, a few other details could have been added. For example the sex distribution of the participants. Also the educational status, current occupation would indicate how tech savvy the patients would have been to participate in tele-health consultations.

The gender distribution of participants had already been indicated in Table 1 heading (Male/Female: 60%/40%). Unfortunately, no additional information on educational status and occupation of patients is available; a mention to this fact was added in the manuscript.

6. The fact that this is a mixed method study with a qualitative component (the last open ended question of the questionnaire) the responses are ideally described as themes, sub themes and specific quotes. Also in the methodology section, there is paucity of information as to how this qualitative data was analysed.

Additional information has been provided.

7. The manuscript lacks a limitations section- even the best of studies will have its own limitations. It needs to be acknowledged.

A limitations paragraph has been added in the Discussion section.

8. In the discussion section, the generisability of the results need to be discussed. If similar results are expected in other parts of Italy or around the world.

Additional comment has been provided in Discussion section.

9. Is there any specific reason why only type 2 diabetics were chosen and not any other types of diabetics?

We considered only the type 2 diabetes, in order to assure homogeneity of patient characteristics and in consideration of different cure management between type 2 and others type of diabetes.

10. The discussion seems to be more focused on BMI and the perception. But it is to be emphasised that there was a lack of statistical significance between the two, though there seemed to be a similar trend.

We agree that conventional correlation (Spearman method) did not reach a statistically significant value regarding BMI and perception. However, the discussion regarding BMI was supported mainly by the results of the principal component analysis (PCA). This technique is a non-parametric method, that can give a general overview of underlying correlations that exist in a set of variables, rather than focusing on a specific single couple of variables. The findings suggested by the biplot from PCA (Figure 3) permits to enucleate the role of BMI with respect also to interactions with all the other variables.

We clarified better this fact in the manuscript.
