# Supplementary material for: Telemedicine and its acceptance by patients with type 2 diabetes mellitus at a single care center during the COVID-19 emergency: A cross-sectional observational study
Source: PLoS One. 2023 Feb 15;18(2):e0269350. doi: 10.1371/journal.pone.0269350 (PMC9931089; doi:10.1371/journal.pone.0269350)
Supplement: Attachment — Submitted filename: Response to Reviewers.docx [file pone.0269350.s002.docx]

Reviewer #3: Dear authors,
Thank you for addressing the queries / suggestions. I suggest that the following be clarified in the manuscript.
1. Being a cross-sectional study, the words "prospective" and "consecutive" can be removed. They are usually used in the context of a prospective cohort study. From your methodology section, it appears that you have chosen all 254 patients who attended the practice within the given time period.

We thank the Reviewer for suggestion. We deleted the words not appropriate for the study, and changed also STROBE diagram in order to make clearer the design of the survey.

2. In the STROBE diagram, there have been 2463 patients between March and May 2020, however only 254 between July and August 2020. Is there a reason why the numbers dramatically reduced?

The STROBE diagram was made clearer; the total number of televisits in the time intervals March - May 2020, corresponding to lockdown period, were 2463; among patients attending televisits, the survey recruited 250 patients who agreed to respond to the questionnaire.

3. As raised by one of the other reviewers, whether Telehealth consultation refers to a video call/consultation or just an audio telephonic call with email communication needs to be clarified better.

Telehealth consultation consisted of a telephone call. The information was explained better in the text.
